# Supplementary material for: Early antibody responses associated with survival in COVID19 patients
Source: PLoS Pathog. 2021 Jul 19;17(7):e1009766. doi: 10.1371/journal.ppat.1009766 (PMC8321400; doi:10.1371/journal.ppat.1009766)
Supplement: S1 Text — Statistical Modeling; Assay Characteristics Fig A, B, and C; Alternative Analyses: IgG Levels at Week 2 and After Week 2 Post-onset Comparisons Fig D, E and Table A; IgG Analysis Using IgG Cut-point MFI Method Fig F; IgG Cut-point MFI Week 1 Post-onset Comparison Fig G; IgG Cut-point MFI Modeling Table S2, Fig H; IgM Analysis: Anti-SARS CoV2 IgM Antibody Kinetics Fig I; IgM Week 1 Post-onset Comparison Fig J; IgM Modeling Table S3, Fig K; IgA Analysis: Anti-SARS CoV2 IgA Antibody Kinetics Fig L; IgA Week 1 Post-onset Comparison Fig M; IgA Modeling Table S4, Fig N; IgG Sensitivity Analysis (exclude 1000 and 1007, A and C in data table): Anti-SARS CoV2 IgG Sensitivity Analysis Kinetics Fig O; IgG Sensitivity Analysis MFI Week 1 Comparison Fig P; IgG Sensitivity Analysis Modeling Table S5, Fig Q; IgG Sensitivity Analysis (Males): Anti-SARS CoV2 IgG Sensitivity Analysis Kinetics Fig R; IgG Sensitivity Analysis MFI Week 1 Comparison Fig S; IgG Sensitivity Analysis Modeling Table S6, Fig T; IgG Covariate Adjusted Analysis Tables G-I; Anti-SARS CoV2 IgG Kinetics–Individual Line Plots Fig U and V. (DOCX) [file ppat.1009766.s002.docx]

**Supplementary Materials**

Contents

[**Statistical Modeling** 2](#_Toc71580102)

[**Model 1** 2](#_Toc71580103)

[**Model 2** 3](#_Toc71580104)

[**Assay Characteristics** 4](#_Toc71580105)

[**IgG Levels at Week 2 and After Week 2 Post-onset Comparisons** 7](#_Toc71580106)

[**IgG Analysis Using IgG Cut-point MFI Method** 9](#_Toc71580107)

[**IgG Cut-point MFI Week 1 Post-onset Comparison** 10](#_Toc71580108)

[**IgG Cut-point MFI Modeling** 10](#_Toc71580109)

[**IgM Analysis** 12](#_Toc71580110)

[**Anti-SARS CoV2 IgM Antibody Kinetics** 12](#_Toc71580111)

[**IgM Week 1 Post-onset Comparison** 13](#_Toc71580112)

[**IgM Modeling** 13](#_Toc71580113)

[**IgA Analysis** 15](#_Toc71580114)

[**Anti-SARS CoV2 IgA Antibody Kinetics** 15](#_Toc71580115)

[**IgA Week 1 Post-onset Comparison** 16](#_Toc71580116)

[**IgA Modeling** 16](#_Toc71580117)

[**IgG Sensitivity Analysis (exclude 1000 and 1007)** 18](#_Toc71580118)

[**Anti-SARS CoV2 IgG Sensitivity Analysis Kinetics** 18](#_Toc71580119)

[**IgG Sensitivity Analysis MFI Week 1 Comparison** 19](#_Toc71580120)

[**IgG Sensitivity Analysis Modeling** 19](#_Toc71580121)

[**IgG Sensitivity Analysis (Males)** 21](#_Toc71580122)

[**Anti-SARS CoV2 IgG Sensitivity Analysis Kinetics** 21](#_Toc71580123)

[**IgG Sensitivity Analysis MFI Week 1 Comparison** 22](#_Toc71580124)

[**IgG Sensitivity Analysis Modeling** 22](#_Toc71580125)

[**IgG Covariate Adjusted Analysis** 24](#_Toc71580126)

[**Anti-SARS CoV2 IgG Kinetics – Individual Line Plots** 25](#_Toc71580127)

## **Statistical Modeling**

### **Model 1**

To examine the relationship between antibody values and outcomes we fit the following linear mixed model for Antibody Response at time $j$ for patient $i$ with outcome $Outcome_{i}$,

$${Antibody Response}_{ij}=\beta_{0}+\beta_{1}I\left( j=Week_{2} \right)+ \beta_{2}I\left( j=Beyond Week_{2} \right)+ I\left( Outcome_{i}=Expired \right)*\left[ \beta_{3}+ \beta_{4}I\left( j=Week_{2} \right)+ \beta_{5}I\left( j=Beyond Week_{2} \right) \right]+b_{i}+\epsilon_{ij}$$

where $I(.)$ is an indicator function that takes the value $1$ if true and $0$ if false.

In the above model, $\beta_{0}$ and $\beta_{3}$ represent the mean response (i.e., antibody titer value) at Week 1 for discharged and expired patients, respectively. The mean increase from Week 1 to Week 2, and Week 1 to after Week 2, is captured by $\beta_{1}$ and $\beta_{2}$, respectively, in discharged patients and by $\beta_{4}$ and $\beta_{5}$, respectively, in expired patients. We account for correlation between observations at different time points for each patient by including a patient-specific random effect $b_{i}$. The random effects for patients are assumed to arise from a normal distribution, i.e., $b_{i}\sim N(0, \tau)$.

### **Model 2**

###

To characterize the relationship between patient-specific antibody response trajectory and eventual outcome, we used a joint modeling approach^[[1]](#footnote-2)^. We first model the antibody response using a linear mixed model, similar to model 1 but without including outcome as a predictor, as follows:

${Antibody Response}_{ij}= \beta_{0}+\beta_{1}I\left( j=Week_{2} \right)+ \beta_{3}I\left( j=Beyond Week_{2} \right)+b_{i0}+ b_{i1}I\left( j=Week_{2} \right)+ b_{i2}I\left( j=Beyond Week_{2} \right)+ \epsilon_{ij}$

Here, random effects $b_{i0}, b_{i1},b_{i2}$ represent patient-specific deviations from the population mean of response at Week 1, increase from Week 1 to Week 2, and Week 1 to after Week 2, respectively. The estimates of the patient-specific deviations, $\hat{b}_{i0}, \hat{b}_{i1},\hat{b}_{i2}$, obtained from fitting the above model are then used as predictors in a probit regression model to predict the eventual outcome as follows:

$Prob\left( Outcome_{i}=Expired \right)=\phi[\gamma_{0}+\gamma_{1}\hat{b}_{i0}+ \gamma_{2}\hat{b}_{i1}+ {\gamma_{3}\hat{b}}_{i2}$]

Here, $\phi$ denotes the cumulative distribution function of the standard normal distribution. The discriminative ability of the model is measured using the area under the receiver operating characteristic curve (AUC).

## **Assay Characteristics**


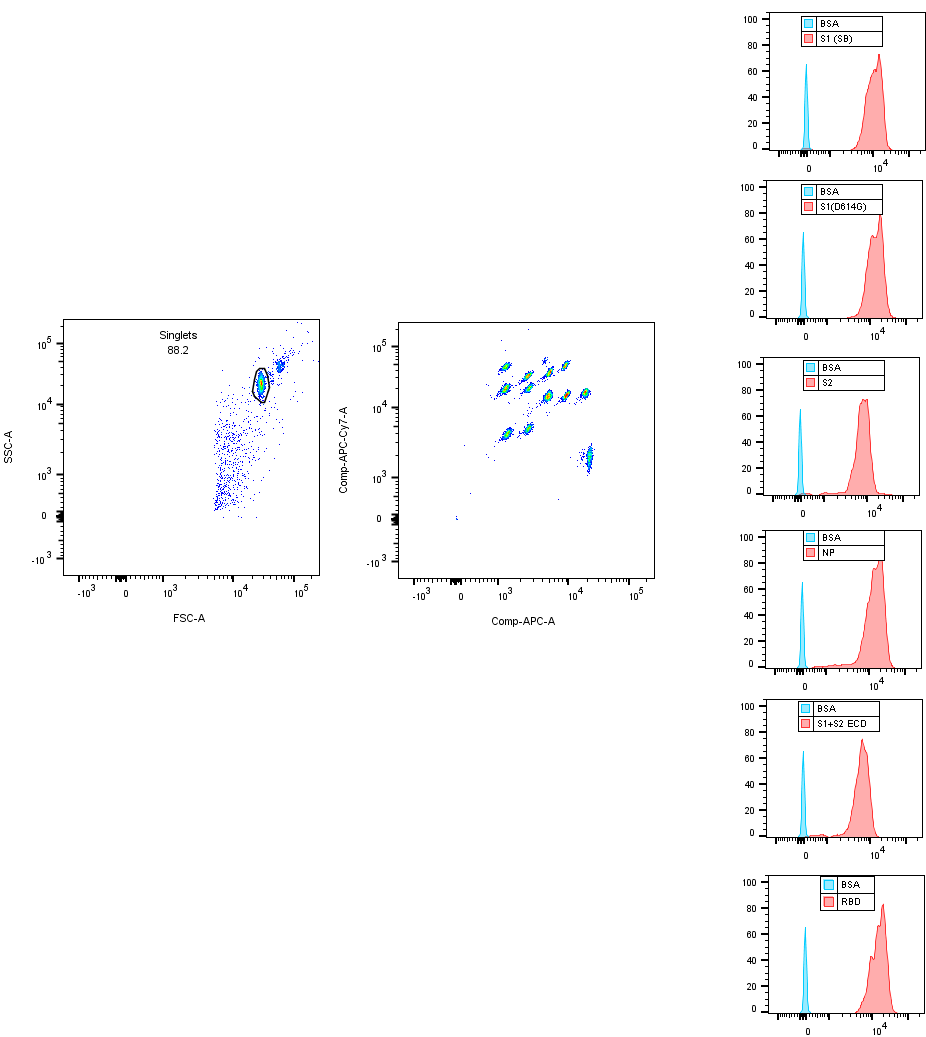


**Fig A Multiplexed beads array for COV2 antibody and flow cytometry analysis.** The singlet beads population were gated based on FSC-SSC display, followed by compensated APC-APC-Cy7 display to show different SARS-CoV2 antigen component-coated target and BSA-control beads. Histograms display overlays of antibody binding signals to antigen-target beads vs BSA beads as indicated.

**Assay Sensitivity and Specificity**

**Fig B Assay Sensitivity and Specificity.** Serum from a COVID19 patient (17G, left side of panels) and a pre-COVID control sample (right side of panels) were incubated with an array of SARS-CoV1 and CoV-2 antigen coated beads, in the presence of free antigens or BSA as indicated. Specific binding signals (MFI) were compared indicating 17G but not the pre-COVID sample contained specific IgG antibodies to COV2-RBD, COV2-N, COV2-S1+S2 (ECD), and cross-reactive to CoV1-RBD and CoV1-S1 (with much less signal intensity as compared to CoV2 antigens). The signals could be inhibited by free antigens as coated on the target beads or free antigens with overlapping epitopes as coated on the target beads.

**Antibody Titer calculation with on-plate standard curve**

**Fig C Example of MFI vs dilution factor chart for antibody to RBD and BSA
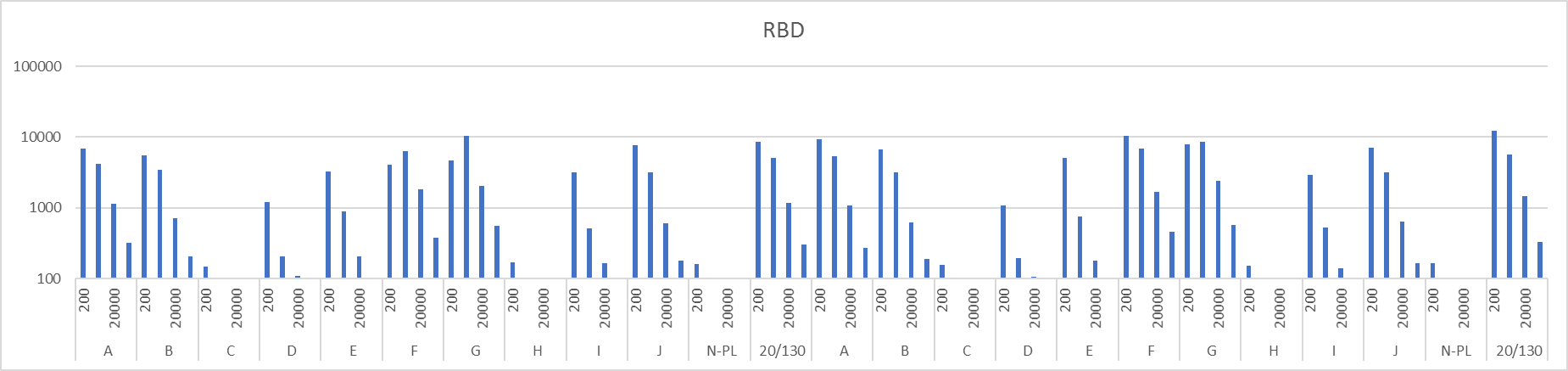
**


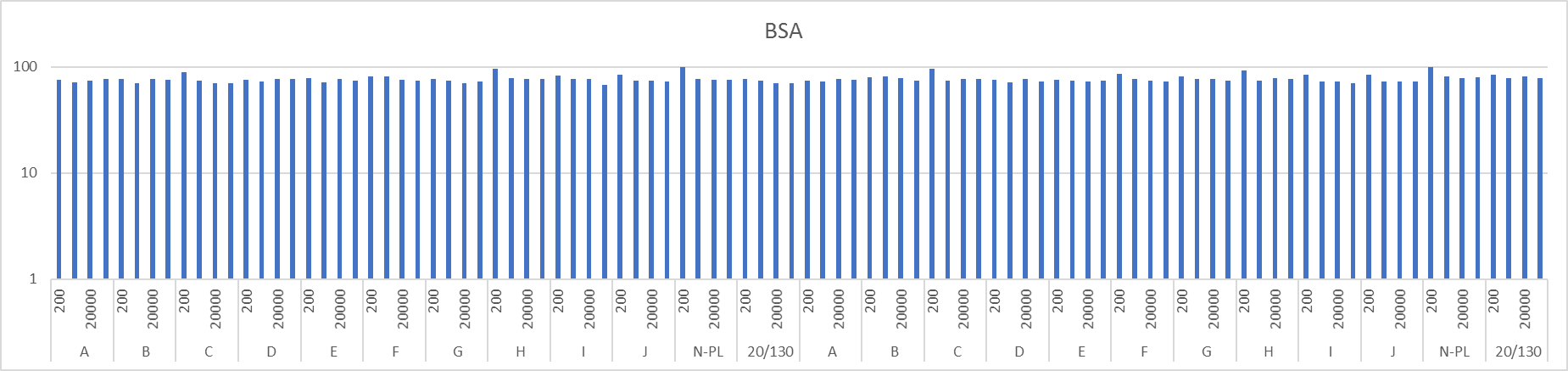


Sample B was chosen as on-plate assay standard to calculate and normalize titer values of all the samples on the same plate (Threshold was at MFI=205)


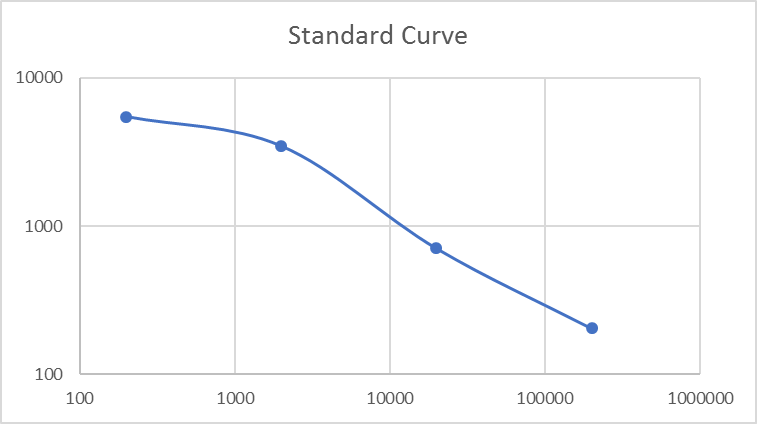

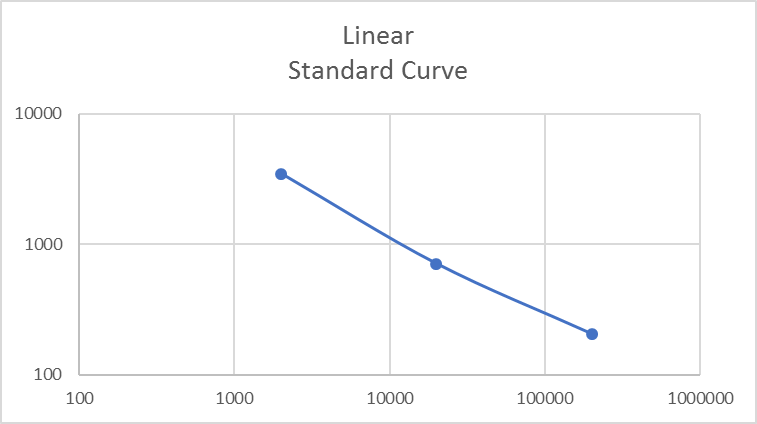


Calculated anti-RBD antibody titer value based on linear regression extrapolation

| Sample | A | B | C | D | E | F | G | H | I | J | N-PL | 20/130 |
| --- | --- | --- | --- | --- | --- | --- | --- | --- | --- | --- | --- | --- |
| Titer | 215675 | 133990 | 0 | 5759 | 25507 | 349687 | 709409 | 0 | 17137 | 119137 | 0 | 244130 |

The lowest values detected in study samples are between 3 and 8 depending on the antigen. Values below those levels were treated as zero. To allow for log transformation a “1” was added to all values so zero values became 1 in the modeling.

## **IgG Levels at Week 2 and After Week 2 Post-onset Comparisons**


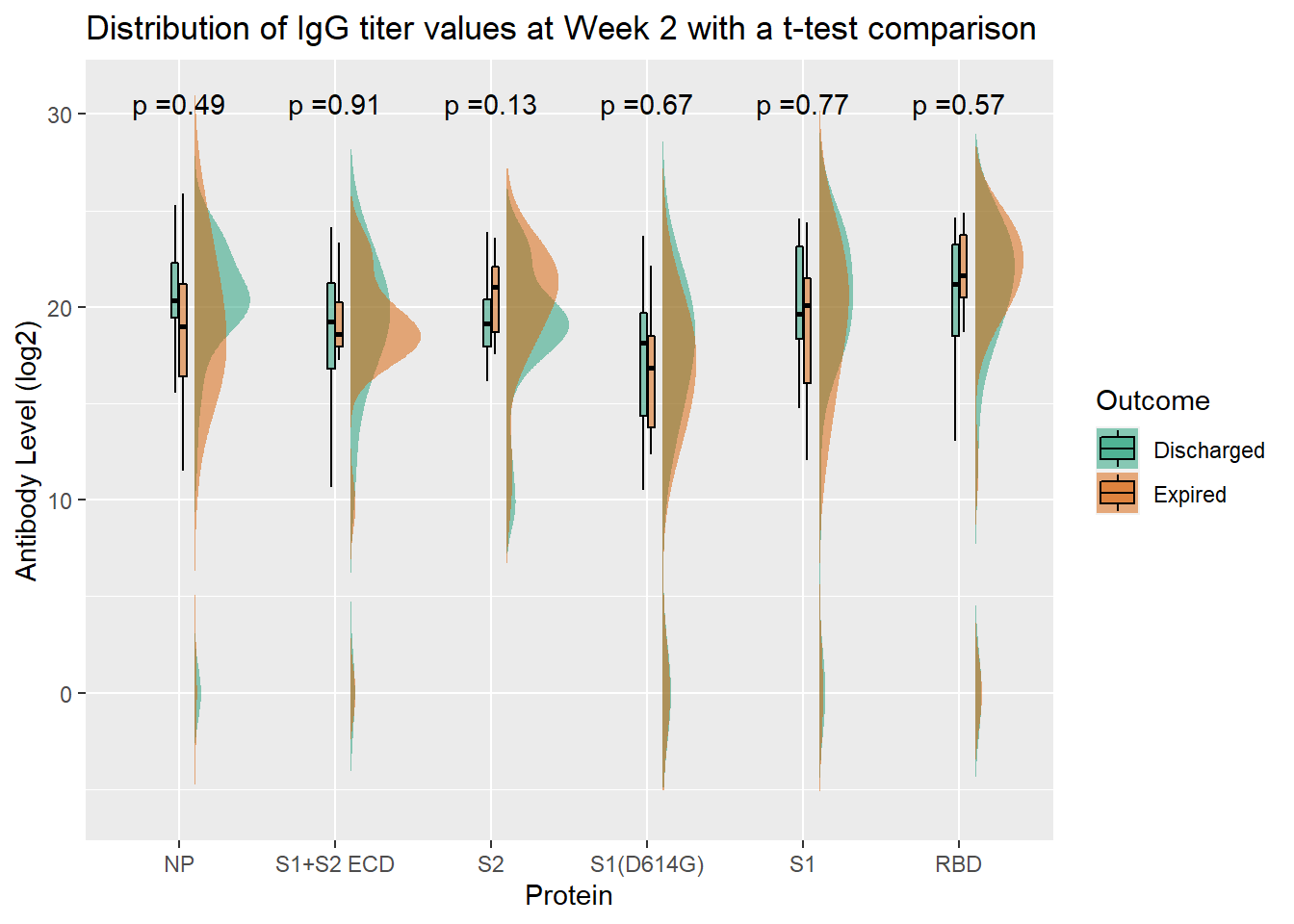


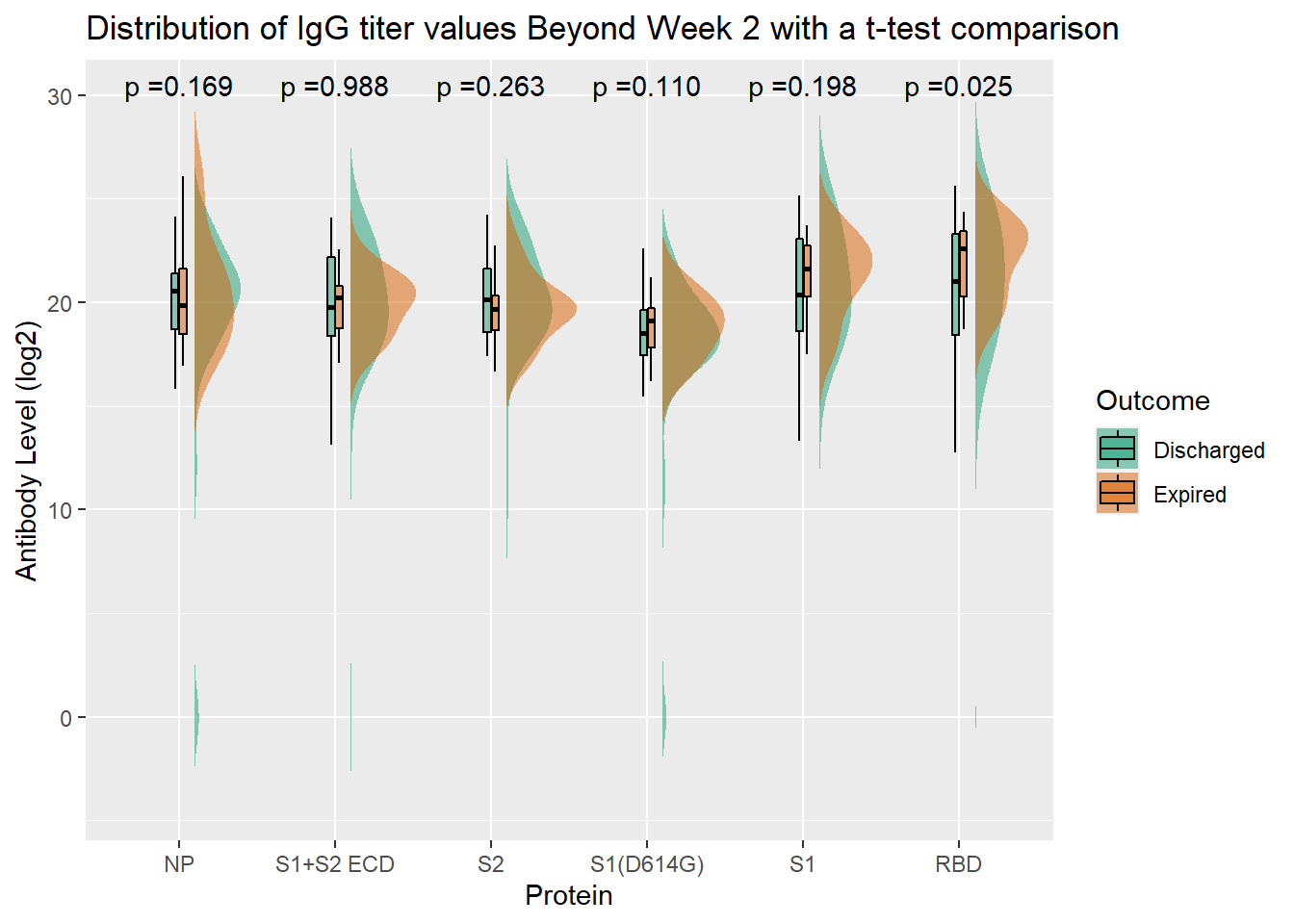


**Fig D** Distribution of IgG antibody levels (log transformed) at week 2 (top) and after week 2 (bottom) post onset in expired and discharged groups with t-test comparison p-value.

**Table A. Estimates from Linear Mixed Model (Model 1) of Difference between Discharged and Expired Groups in other IgG Antibody Levels (log) by Time**

|  | Average Difference between Discharged and Expired groups | | | |
| --- | --- | --- | --- | --- |
|  | NP | S1S2ECD | S1D614 | RBD |
| Week1 | 2.71 | 2.62 | 4.89* | 4.61 |
| Week2 | 2.07 | 0.18 | 1.73 | 1.01 |
| Beyond Week2 | 1.79 | 1.78 | 1.44 | 2.32 |
| Change from week1 to week2 | -0.64 | -2.44* | -3.16 | -3.60 |
| Change from week1 to beyond week2 | -0.92 | -0.84 | -3.46* | -2.29 |
| *Statistically Significant Differences in IgG Titer Values between Discharged and Expired groups at $\alpha=0.05$ | | | | |


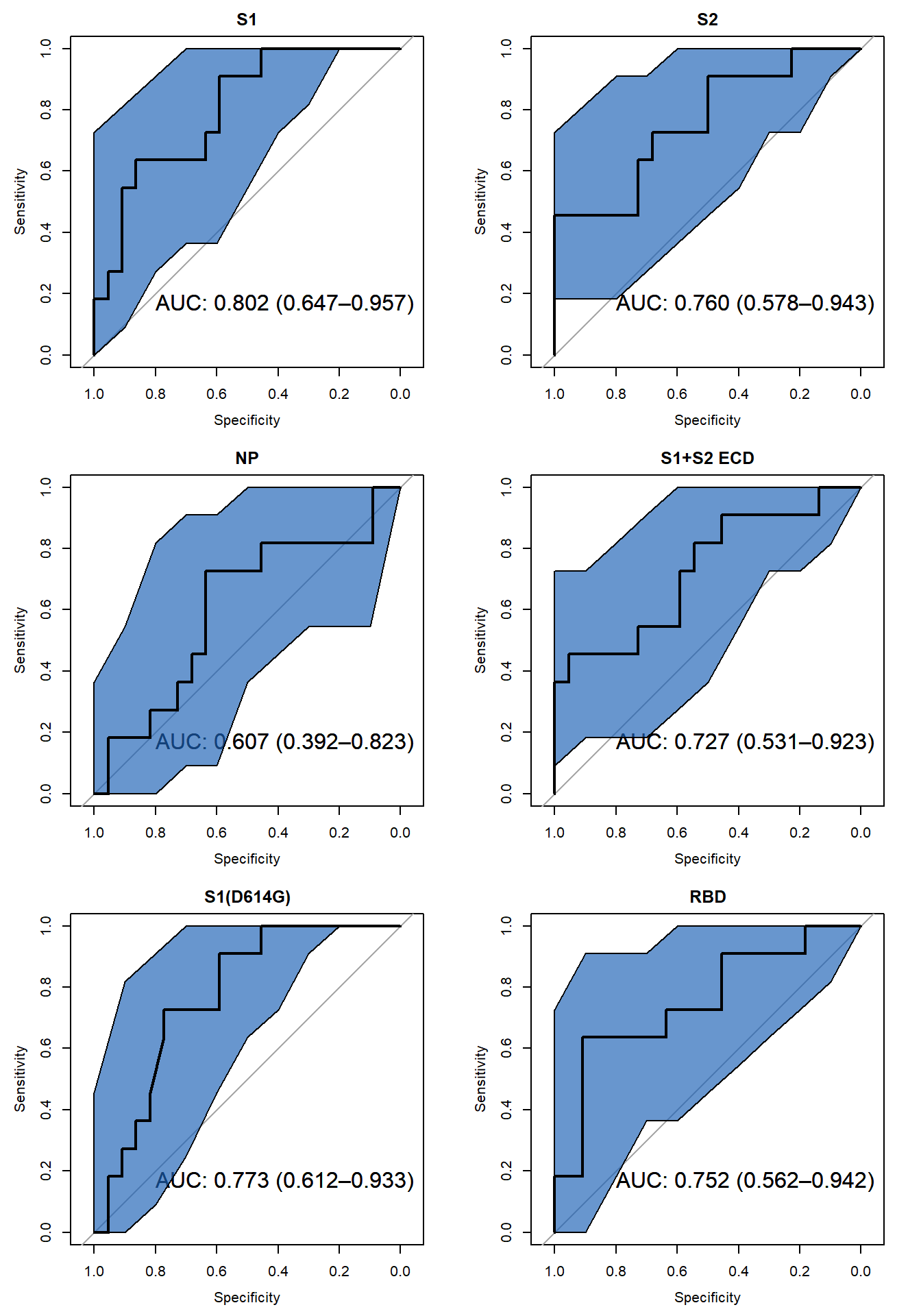


**Fig E Cut-point MFI.** ROC curves for joint model (model 2) to predict death using IgG antibody levels; area under the curve (AUC) is presented within figure with 95% confidence intervals.

## **IgG Analysis Using IgG Cut-point MFI Method**

**Anti-SARS CoV2 IgG Cut-point MFI Antibody Kinetics**

**Analysis with Antibody level expressed by maximum signal of MFI x dilution factor for MFI > 3SD + Pre-COVID baseline**


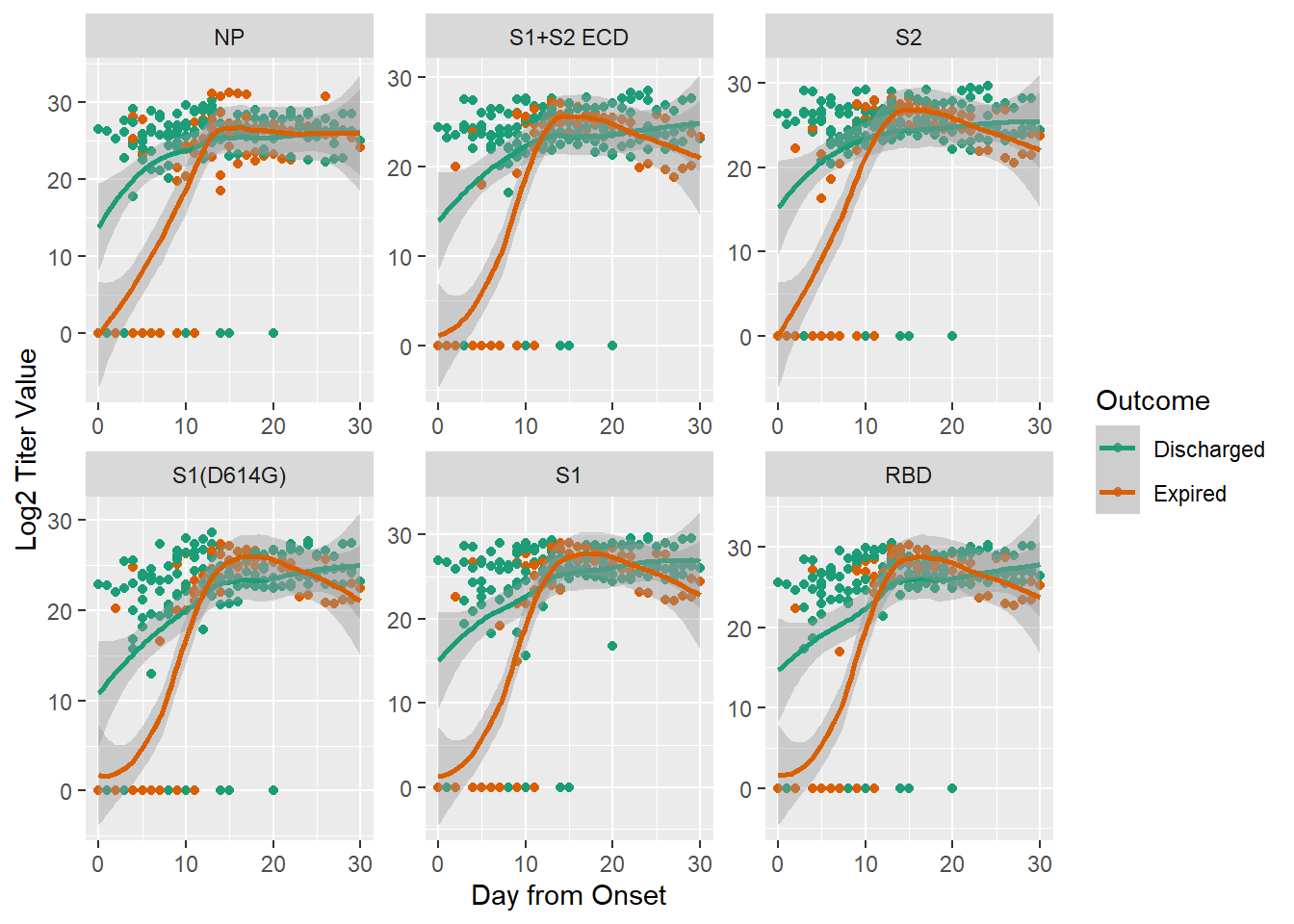


**Fig F Anti-SARS CoV2 IgG Cut-point MFI Antibody Kinetics.** Observed IgG antibody titer values (log transformed) in expired and discharged groups of patients by protein antigen. Points are observed values for each patient at corresponding day from onset; lines are smoothed regression lines fit to the observed data with 95% confidence interval bands.

### **IgG Cut-point MFI Week 1 Post-onset Comparison**


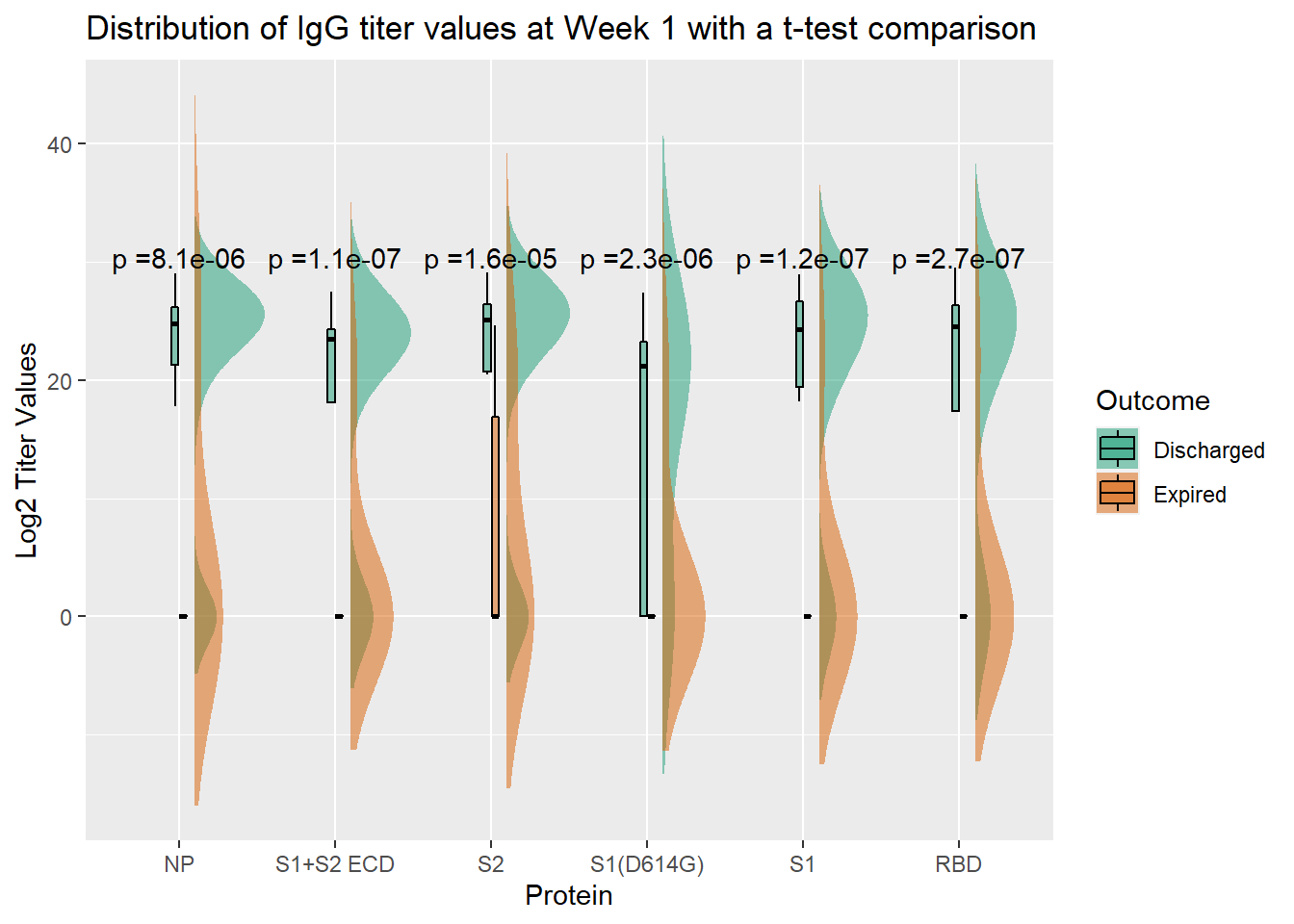


**Fig G Comparison of Week1 (0 to 7 days) After Onset Anti-SARS COV2 IgG Antibody Titers Between Discharged and Expired Groups using IgG Cut-point MFI.** Antibody Distribution of IgG titer values (log transformed) at week 1 post onset in expired and discharged groups with t-test comparison p-value.

### **IgG Cut-point MFI Modeling**

**Table B. Estimates from Linear Mixed Model (Model 1) of Difference between Discharged and Expired Groups in IgG Antibody Levels (log) by Time**

|  | Average Difference between Discharged and Expired groups | | | | | |
| --- | --- | --- | --- | --- | --- | --- |
|  | NP | S1 | S2 | S1S2ECD | S1D614 | RBD |
| Week1 | 9.59* | 11.1* | 8.40* | 10.0* | 7.59* | 9.38* |
| Week2 | 3.75 | 1.98 | 2.90 | 2.69 | 2.08 | 1.83 |
| Beyond Week2 | 1.99 | 3.18 | 2.62 | 1.99 | 2.26 | 2.17 |
| Change from week1 to week2 | -5.84* | -9.12* | -5.50* | -7.30* | -5.51* | -7.55* |
| Change from week1 to beyond week2 | -7.61* | -7.91* | -5.78* | -8.01* | -5.33* | -7.21* |
| *Statistically Significant Differences in IgG Titer Values between Discharged and Expired groups at $\alpha=0.05$ | | | | | | |


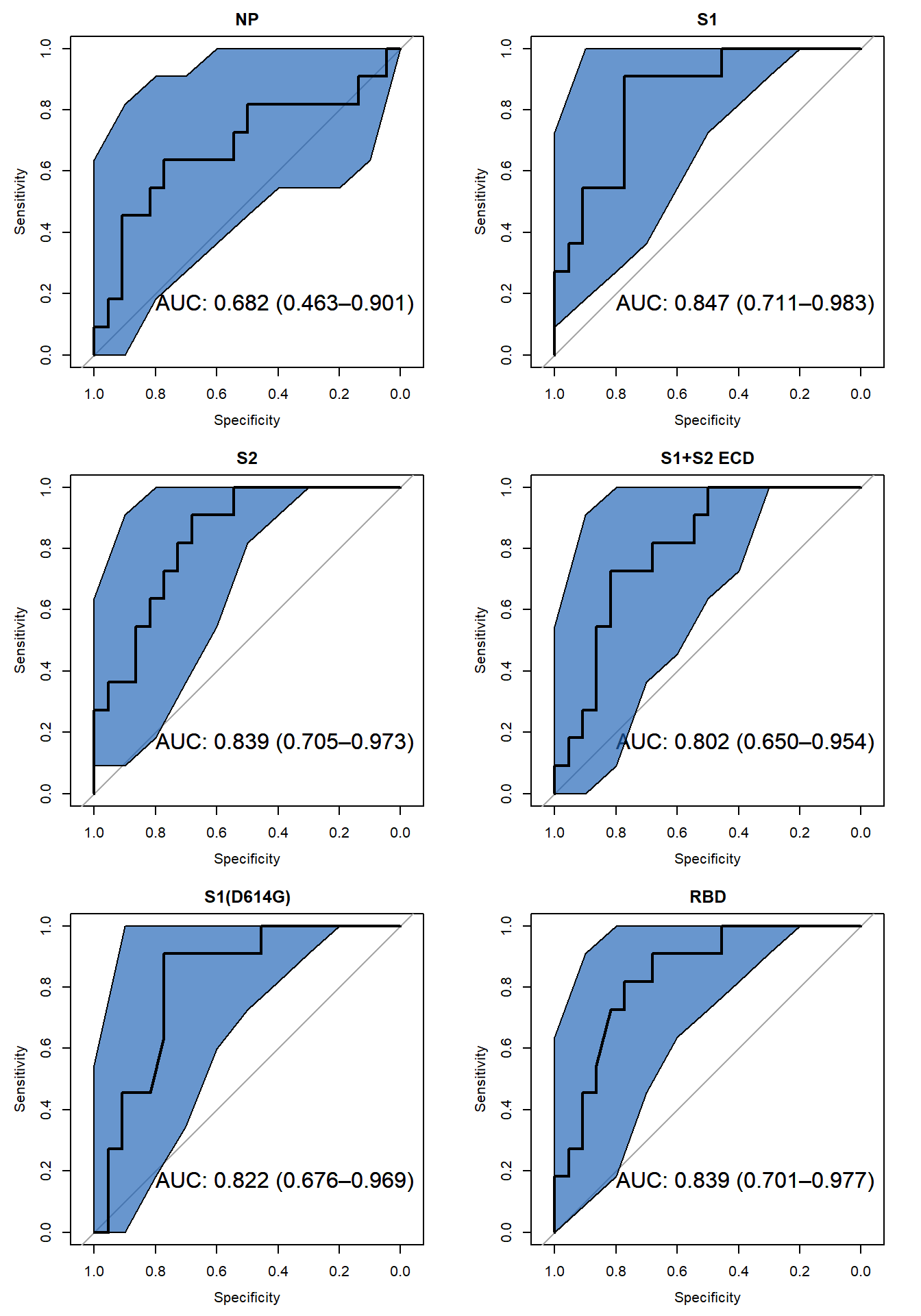


**Fig H Cut-point MFI.** ROC curves for joint model (model 2) to predict death using IgG antibody levels; area under the curve (AUC) is presented within figure with 95% confidence intervals.

## **IgM Analysis**

### **Anti-SARS CoV2 IgM Antibody Kinetics**


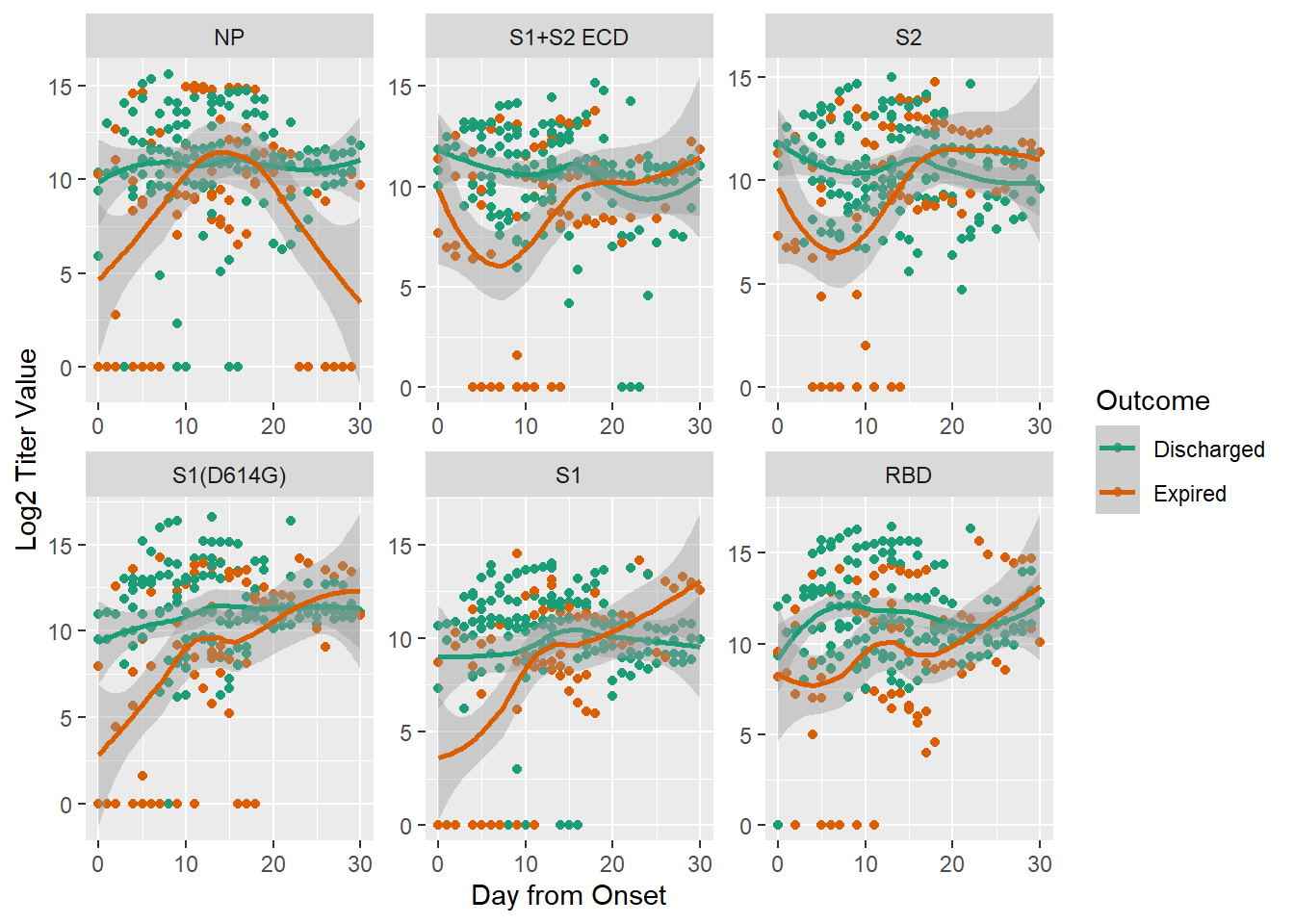


**Fig I** Observed IgM antibody titer values (log transformed) in expired and discharged groups of patients by protein antigen. Points are observed values for each patient at corresponding day from onset; lines are smoothed regression lines fit to the observed data with 95% confidence intervals.

### **IgM Week 1 Post-onset Comparison**


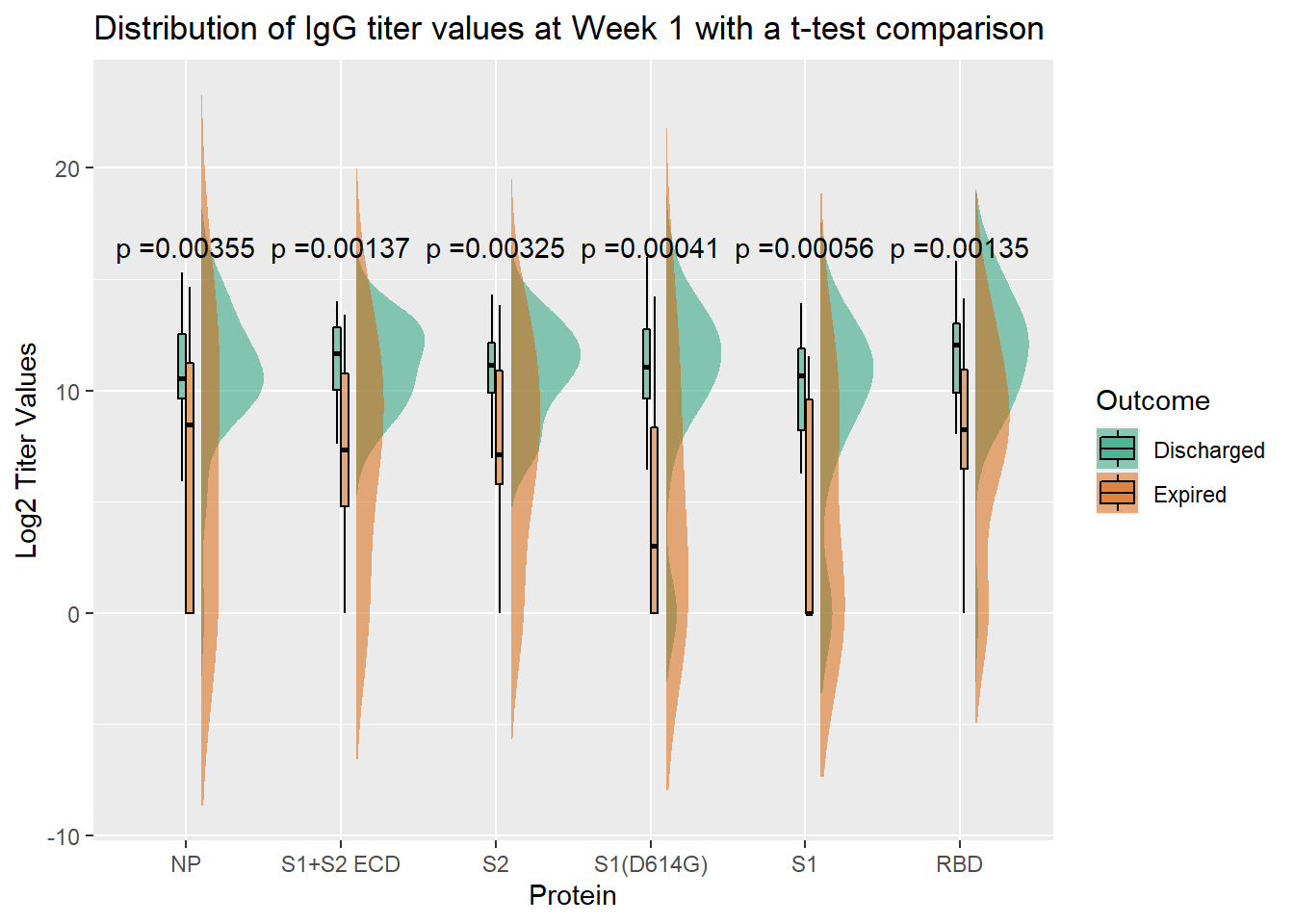


**Fig J** Distribution of IgM antibody levels (log transformed) at week 1 post onset in expired and discharged groups with t-test comparison p-value.

### **IgM Modeling**

**Table C. Estimates from Linear Mixed Model (Model 1) of Difference between Discharged and Expired Groups in IgM Antibody Levels (log) by Time**

|  | Average Difference between Discharged and Expired groups | | | | | |
| --- | --- | --- | --- | --- | --- | --- |
|  | NP | S1 | S2 | S1S2ECD | S1D614 | RBD |
| Week1 | 2.05 | 2.54 | 3.08* | 3.91* | 4.13* | 3.14* |
| Week2 | 0.70 | 0.42 | 1.27 | 1.52 | 1.42 | 1.33 |
| Beyond Week2 | 2.57 | 1.73 | 1.86 | 2.00 | 3.06* | 3.40* |
| Change from week1 to week2 | -1.35 | -2.12* | -1.81* | -2.38* | -2.71* | -1.81* |
| Change from week1 to beyond week2 | 0.52 | -0.81 | -1.22* | -1.91* | -1.07 | 0.27 |
| *Statistically Significant Differences in IgM Titer Values between Expired and Discharged groups at $\alpha=0.05$ | | | | | | |


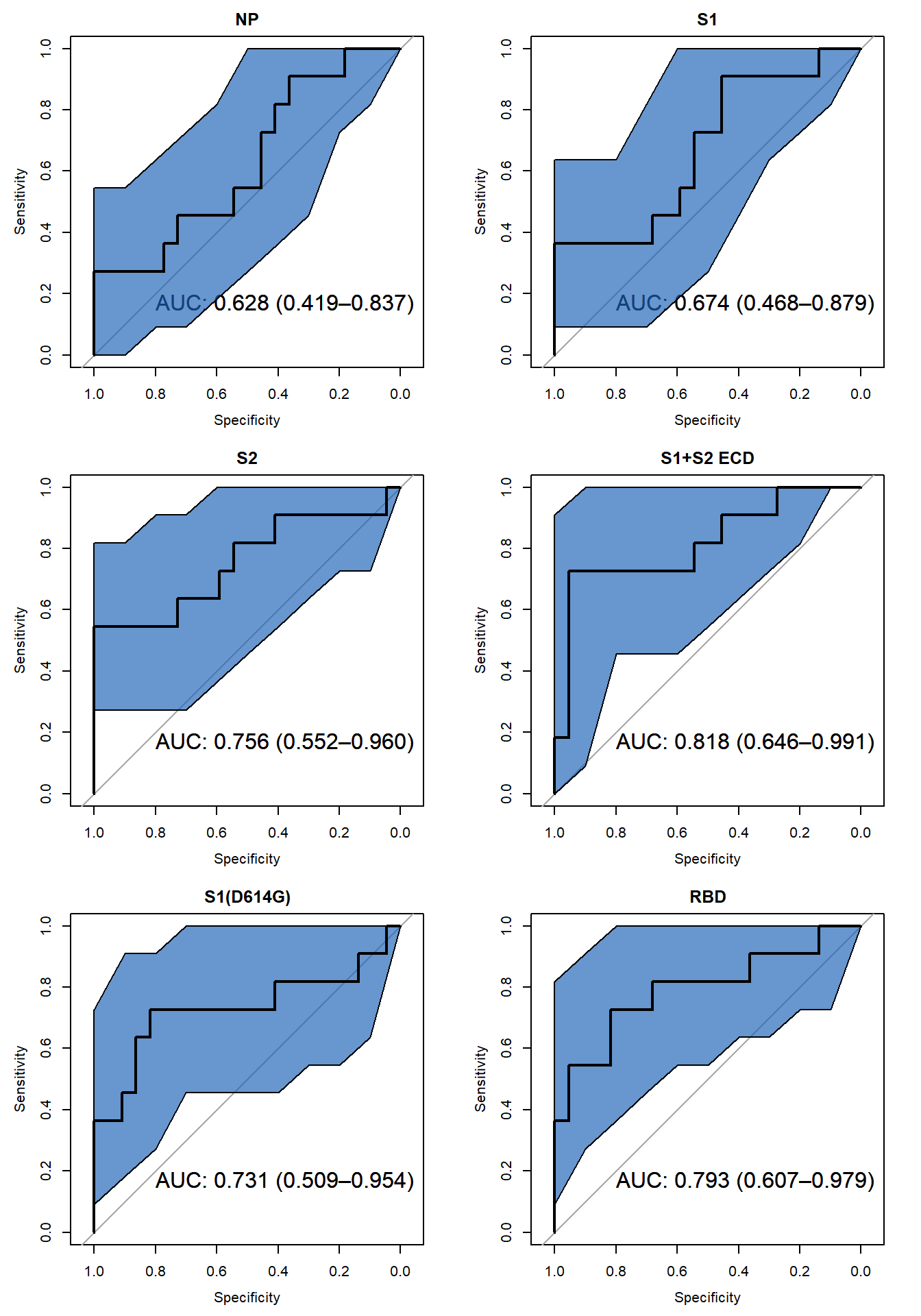


**Fig K** ROC curves for joint model (model 2) to predict death using IgM antibody levels; area under the curve (AUC) is presented within figure with 95% confidence intervals.

## **IgA Analysis**

### **Anti-SARS CoV2 IgA Antibody Kinetics**


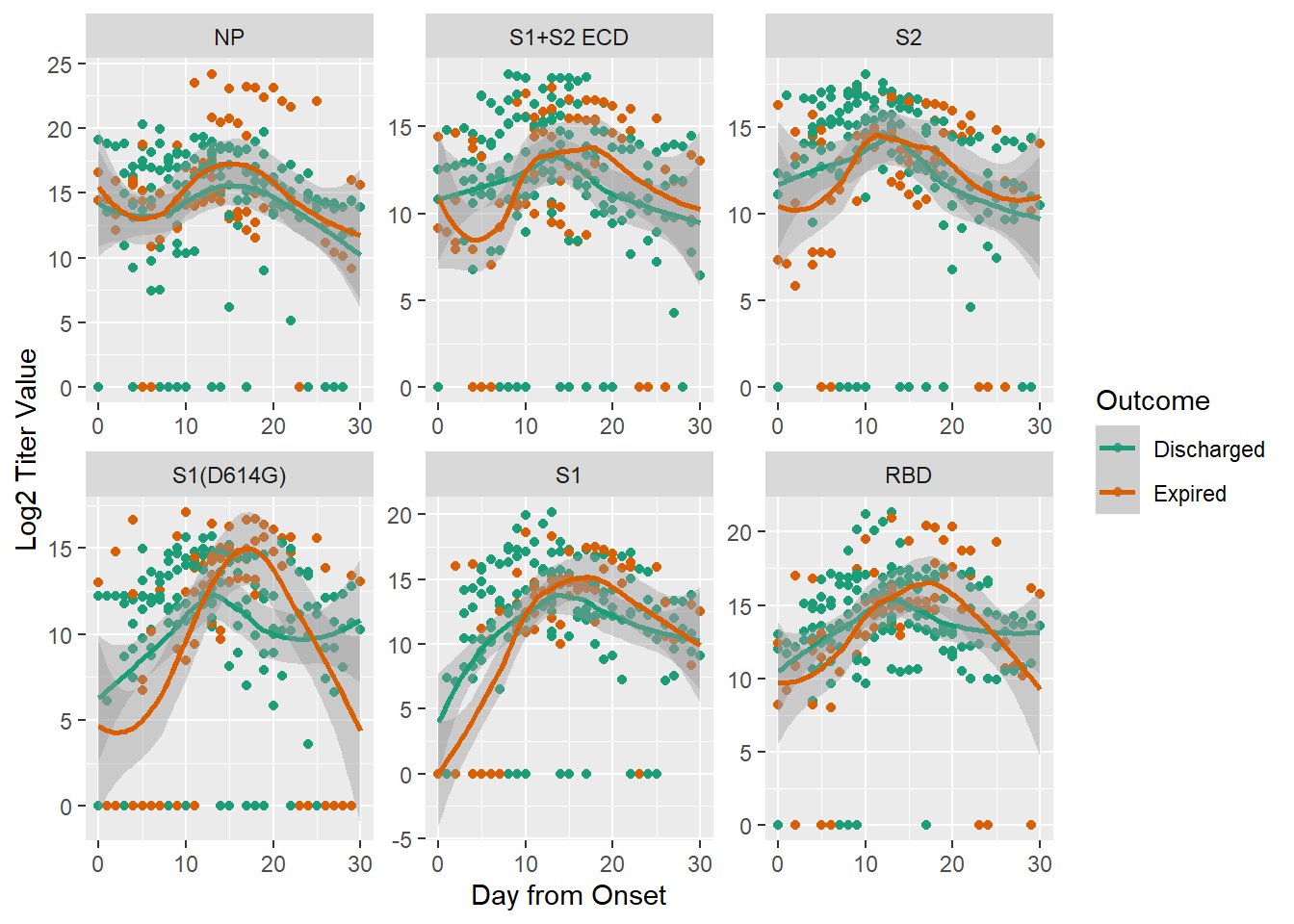


**Fig L** Observed IgA antibody levels (log transformed) in expired and discharged groups of patients by protein antigen. Points are observed values for each patient at corresponding day from onset; lines are smoothed regression lines fit to the observed data with 95% confidence intervals.

### **IgA Week 1 Post-onset Comparison**


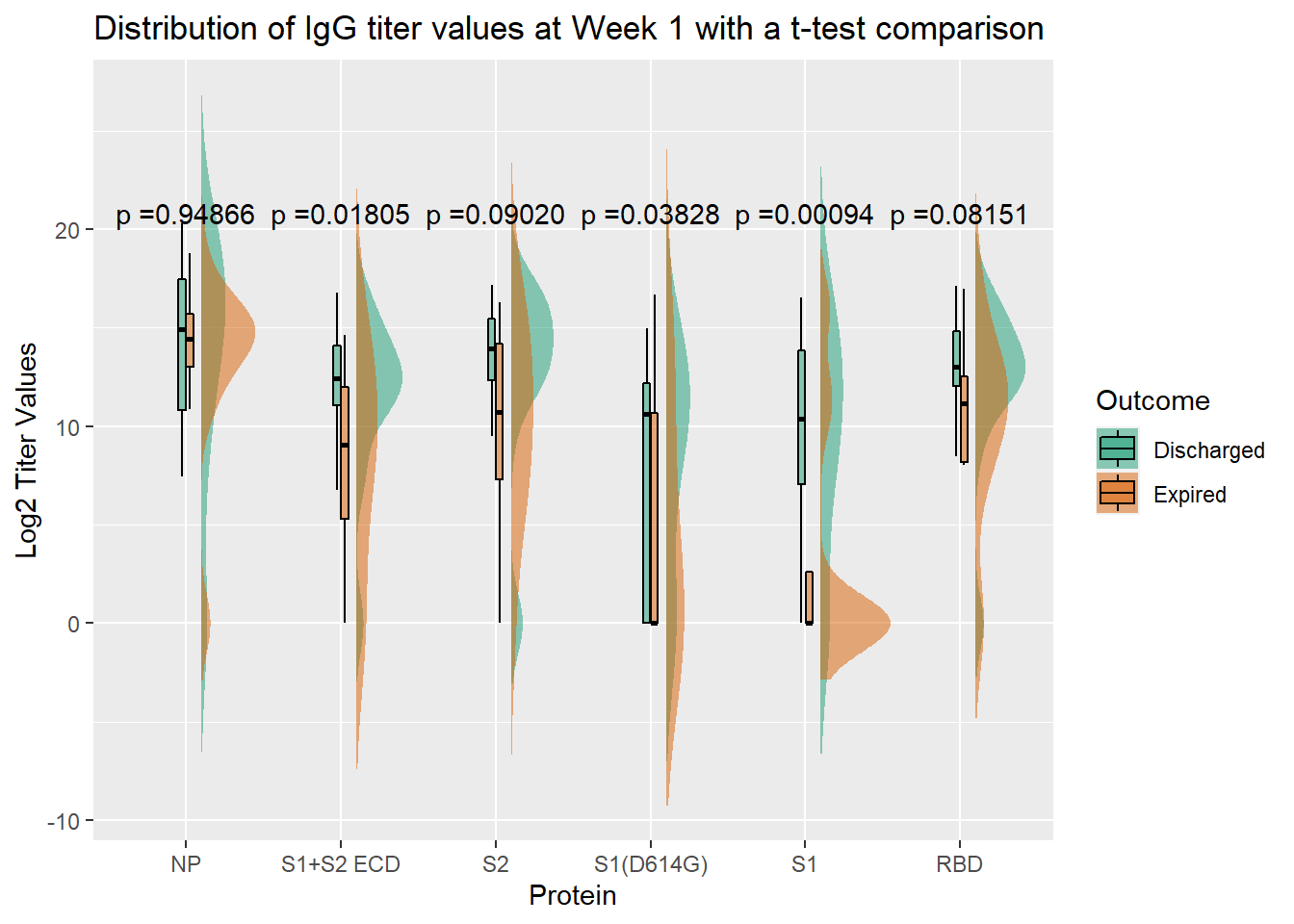


**Fig M** Distribution of IgA antibody levels (log transformed) at week 1 post onset in expired and discharged groups with t-test comparison p-value.

### **IgA Modeling**

**Table D. Estimates from Linear Mixed Model (Model 1) of Difference between Discharged and Expired Groups in IgA Antibody Levels (log) by Time**

|  | Average Difference between Discharged and Expired groups | | | | | |
| --- | --- | --- | --- | --- | --- | --- |
|  | NP | S1 | S2 | S1S2ECD | S1D614 | RBD |
| Week1 | -0.57 | 4.42* | 1.17 | 2.83 | 1.11 | 1.43 |
| Week2 | -2.02 | -1.35 | -1.20 | -1.31 | 0.25 | -0.43 |
| Beyond Week2 | -0.63 | -0.04 | 0.02 | -0.92 | -0.09 | -0.04 |
| Change from week1 to week2 | -1.45 | -5.77* | -2.37 | -4.15* | -0.86 | -1.86 |
| Change from week1 to beyond week2 | -0.06 | -4.46* | -1.15 | -3.75* | -1.19 | -1.48 |
| *Statistically Significant Differences in IgA Titer Values between Expired and Discharged groups at $\alpha=0.05$ | | | | | | |


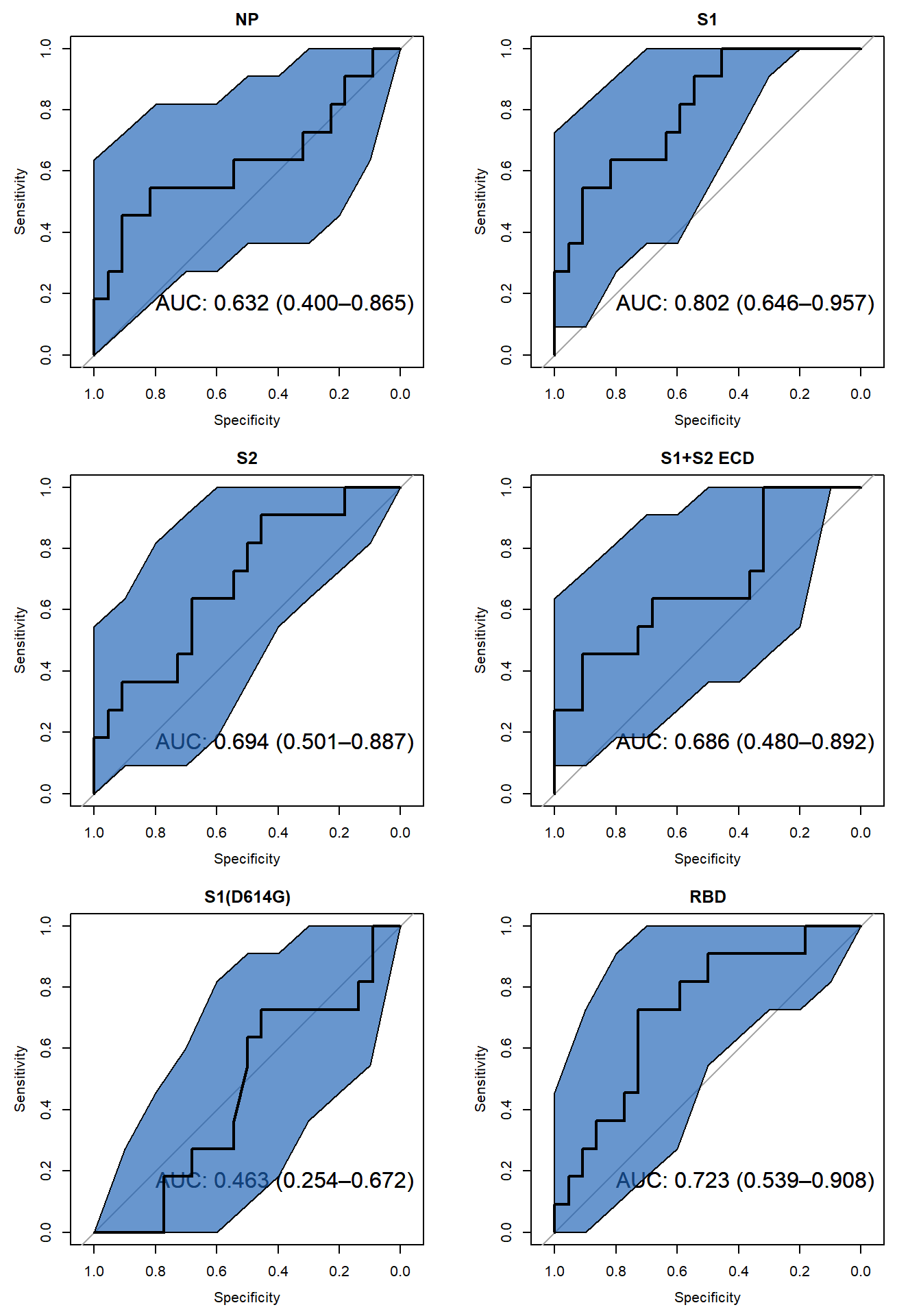


**Fig N** ROC curves for joint model (model 2) to predict death using IgM antibody levels; area under the curve (AUC) is presented within figure with 95% confidence intervals.

## **IgG Sensitivity Analysis (exclude 1000 and 1007)**

For two patients there was a potential misclassification of some samples based on a discrepancy between logs. To rule out any potential impact, analyses were run completely removing both of those patients. Exclusion of those patients had no impact on the results.

### **Anti-SARS CoV2 IgG Sensitivity Analysis Kinetics**


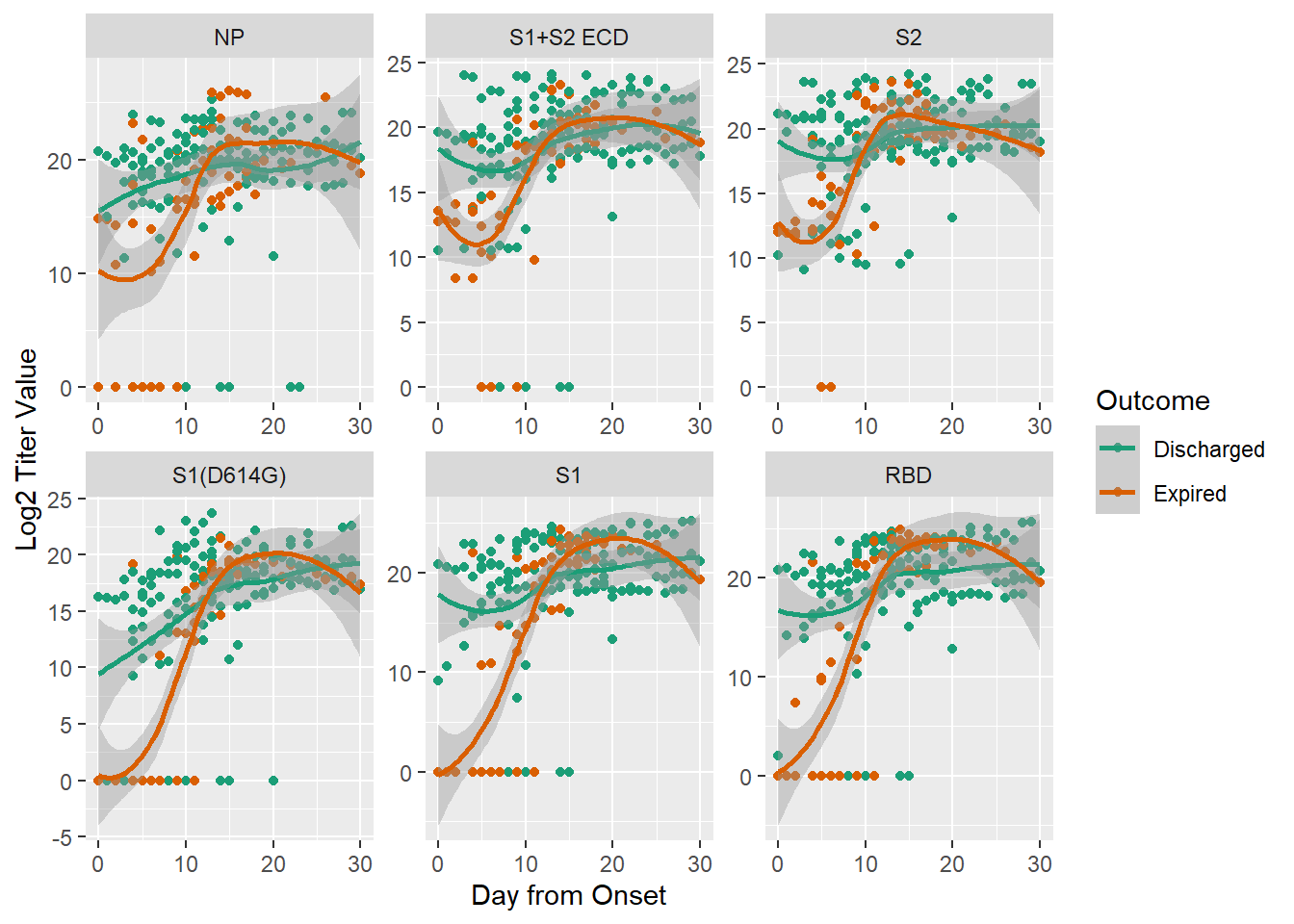


**Fig O Anti-SARS CoV2 IgG Kinetics (exclude 1000 and 1007).** Observed IgG antibody titer values (log transformed) in expired and discharged groups of patients by protein antigen. Points are observed values for each patient at corresponding day from onset; lines are smoothed regression lines fit to the observed data with 95% confidence intervals.

### **IgG Sensitivity Analysis MFI Week 1 Comparison**


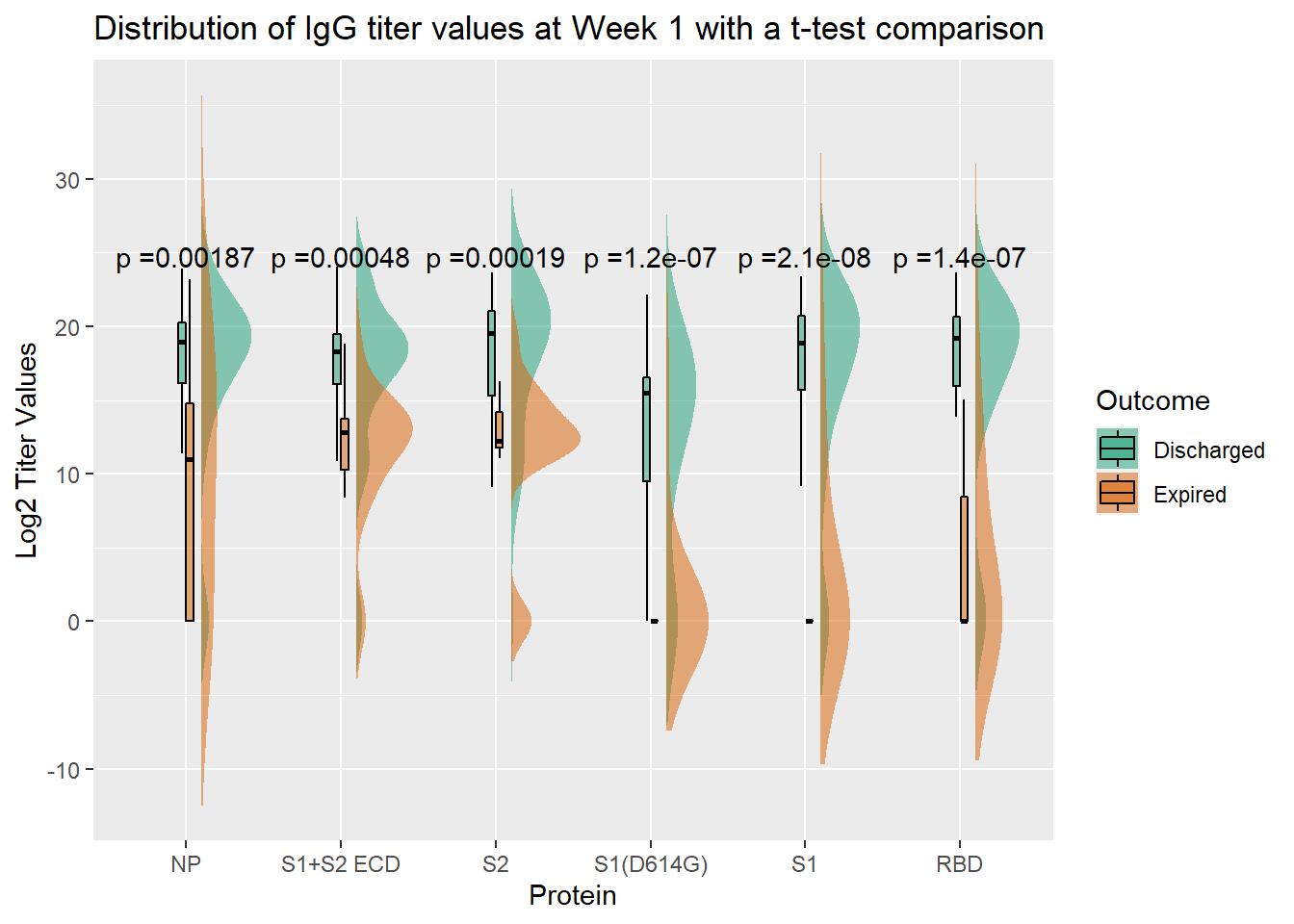


**Fig P IgG Cut-point MFI Week 1 Comparison (exclude 1000 and 1007).** Distribution of IgG antibody levels (log transformed) at week 1 post onset in expired and discharged groups with t-test comparison p-value.

### **IgG Sensitivity Analysis Modeling**

**Table E. Estimates from Linear Mixed Model (Model 1) of Difference between Discharged and Expired Groups in IgG Antibody Levels (log) by Time**

|  | Average Difference between Discharged and Expired groups | | | | | |
| --- | --- | --- | --- | --- | --- | --- |
|  | NP | S1 | S2 | S1S2ECD | S1D614 | RBD |
| Week1 | 2.91 | 8.68* | 4.16* | 3.33 | 6.50* | 6.28* |
| Week2 | 2.03 | 1.97 | 0.13 | 0.32 | 2.06 | 1.25 |
| Beyond Week2 | 1.78 | 2.21 | 1.54 | 1.37 | 1.03 | 2.05 |
| Change from week1 to week2 | -0.88 | -6.72* | -4.02* | -3.00* | -4.45* | -5.03* |
| Change from week1 to beyond week2 | -1.13 | -6.48* | -2.61* | -1.95 | -5.47* | -4.23* |
| *Statistically Significant Differences in IgG Titer Values between Expired and Discharged groups at $\alpha=0.05$ | | | | | | |


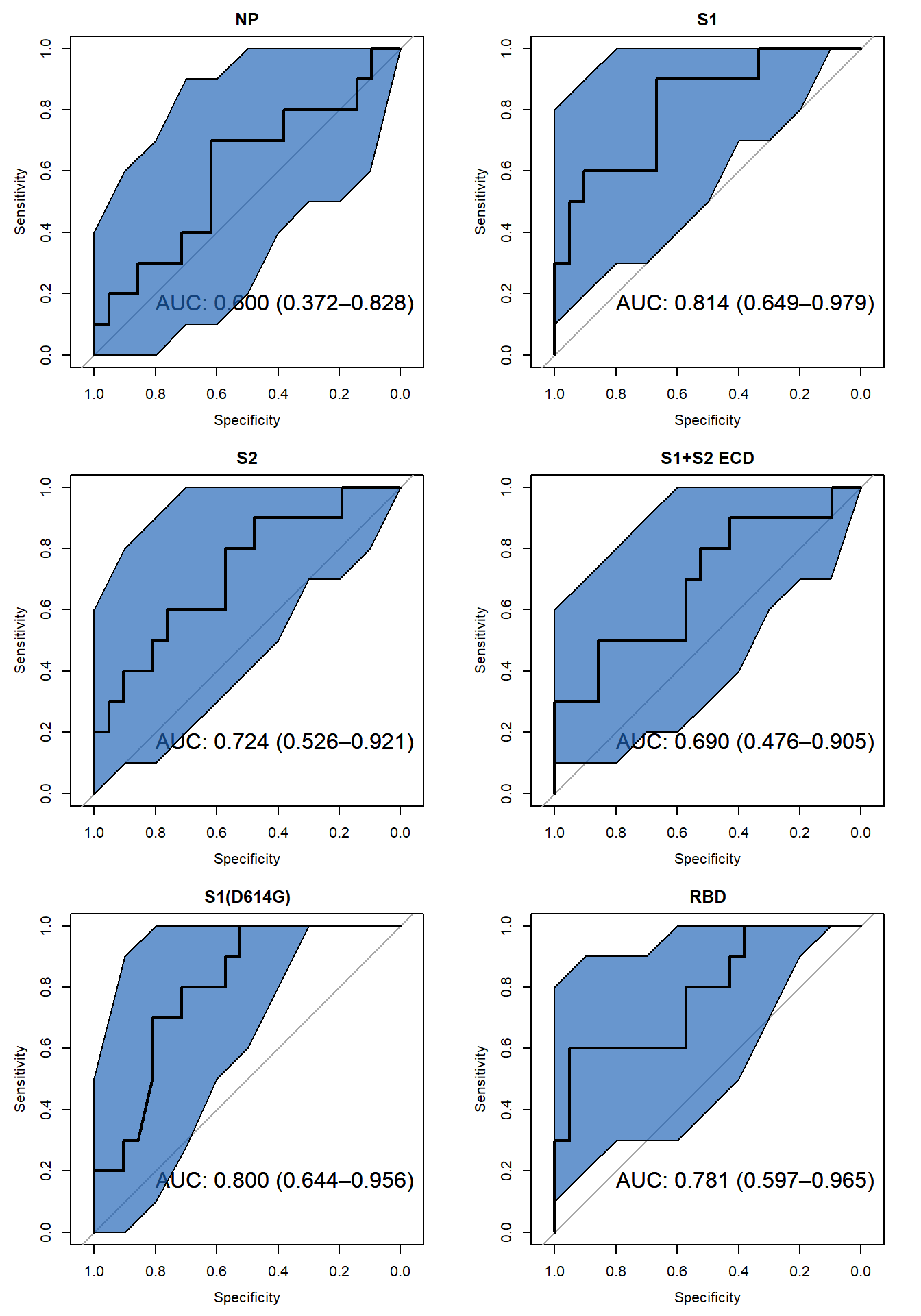


**Fig Q** ROC curves for joint model (model 2) to predict death using IgG antibody levels; area under the curve (AUC) is presented within figure with 95% confidence intervals.

## **IgG Sensitivity Analysis (Males)**

As in the expired group, only one patient out of 11 was female, to rule out any potential gender bias of our finding in discharged and expired groups, analyses were run for only male patients. Exclusion of female patients had no impact on the results.

### **Anti-SARS CoV2 IgG Sensitivity Analysis Kinetics**


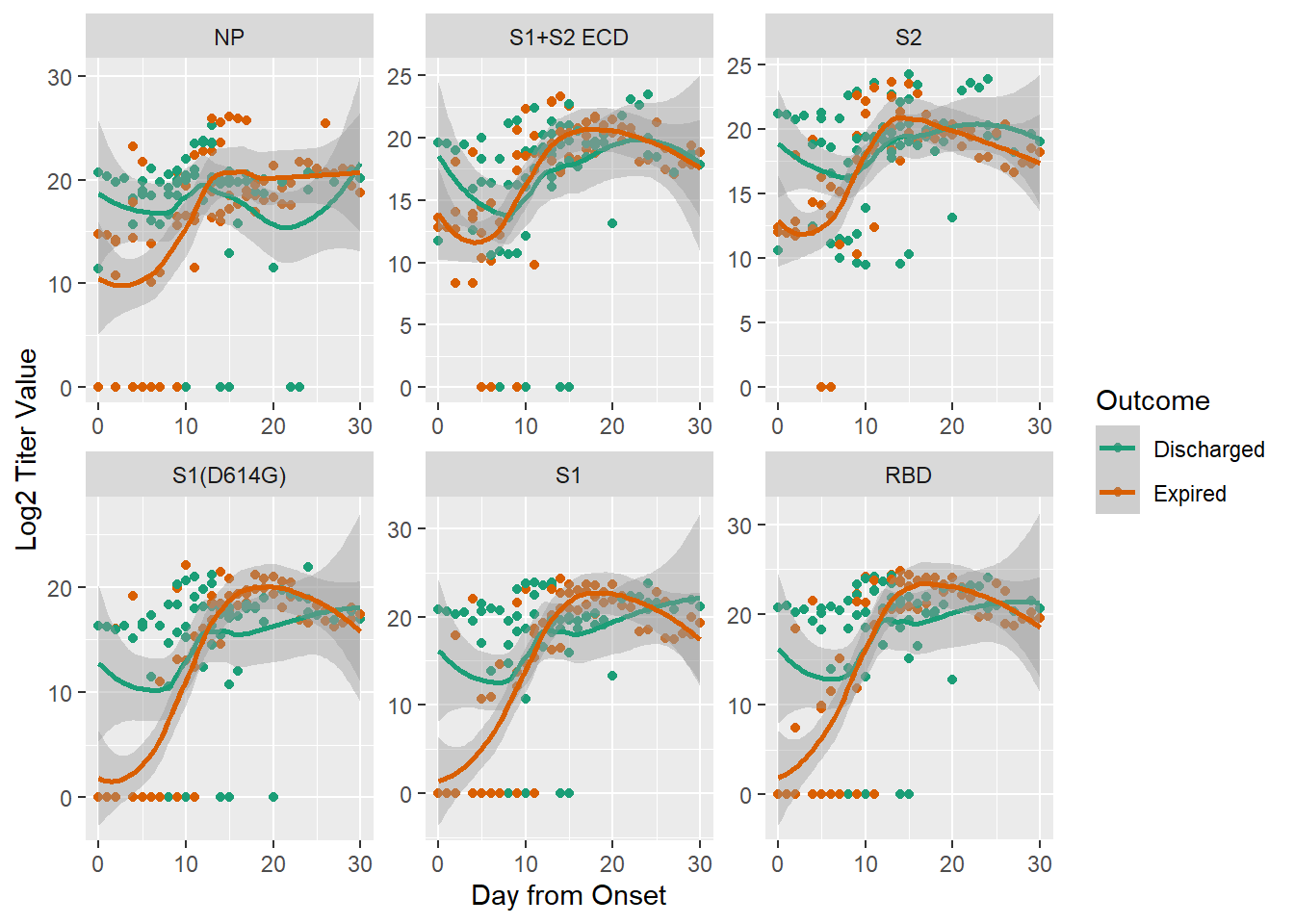


**Fig R Anti-SARS CoV2 IgG Kinetics (Males).** Observed IgG antibody titer values (log transformed) in expired and discharged groups of patients by protein antigen. Points are observed values for each patient at corresponding day from onset; lines are smoothed regression lines fit to the observed data with 95% confidence intervals.

### **IgG Sensitivity Analysis MFI Week 1 Comparison**


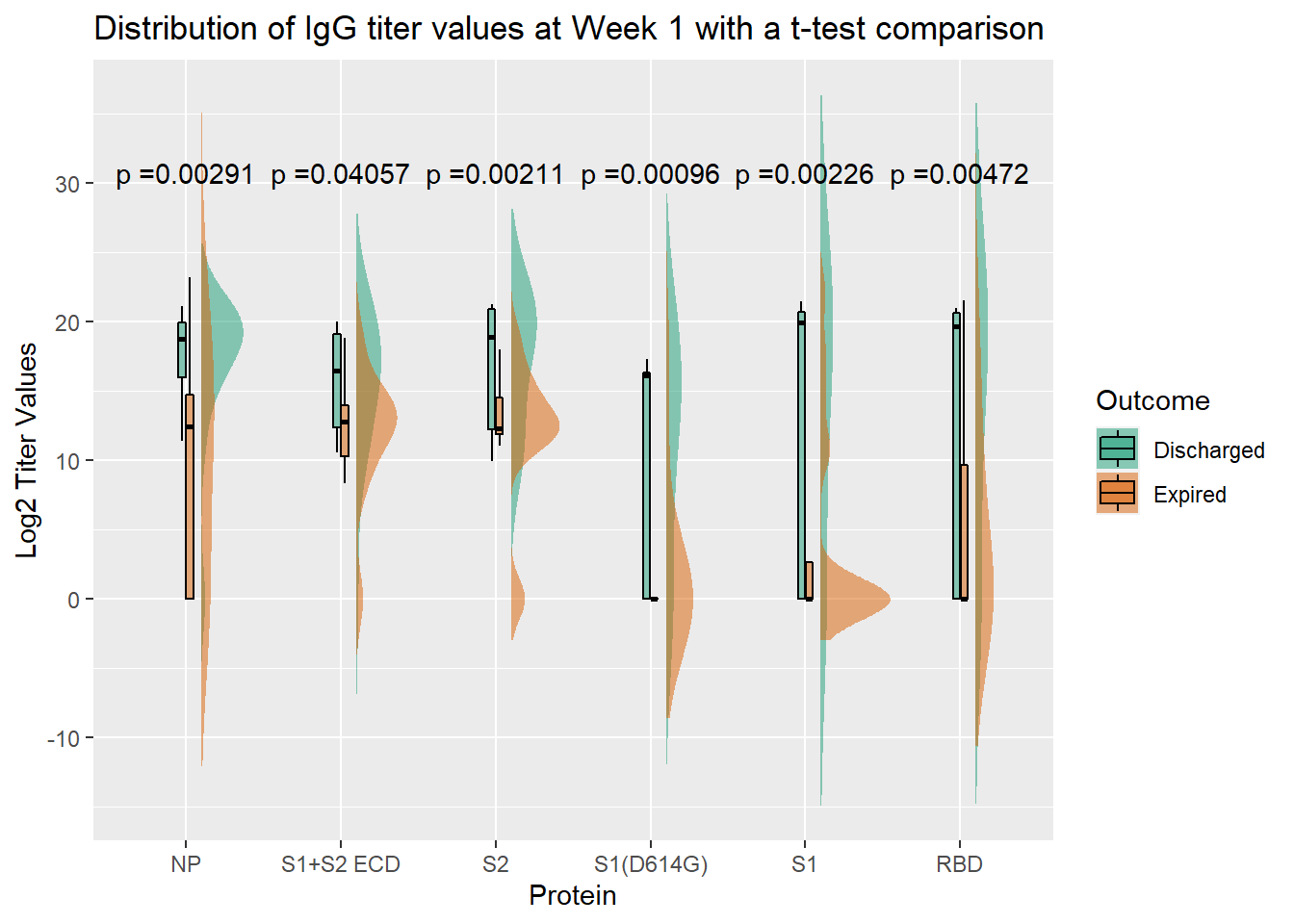


**Fig S IgG Cut-point MFI Week 1 Comparison (Males).** Distribution of IgG antibody levels (log transformed) at week 1 post onset in expired and discharged groups with t-test comparison p-value.

### **IgG Sensitivity Analysis Modeling**

**Table F. Estimates from Linear Mixed Model (Model 1) of Difference between Discharged and Expired Groups in IgG Antibody Levels (log) by Time**

|  | Average Difference between Discharged and Expired groups | | | | | |
| --- | --- | --- | --- | --- | --- | --- |
|  | NP | S1 | S2 | S1S2ECD | S1D614 | RBD |
| Week1 | 2.78 | 5.11 | 3.99* | 2.16 | 4.81 | 2.74 |
| Week2 | 1.27 | 0.96 | -0.22 | -1.46 | 1.04 | 0.45 |
| Beyond Week2 | 1.23 | 1.67 | 0.95 | 0.46 | 0.06 | 1.92 |
| Change from week1 to week2 | -1.51 | -4.14* | -4.21* | -3.62* | -3.77 | -2.29 |
| Change from week1 to beyond week2 | -1.56 | -3.44 | -3.03* | -1.70 | -4.75* | -0.82 |
| *Statistically Significant Differences in IgG Titer Values between Expired and Discharged groups at $\alpha=0.05$ | | | | | | |


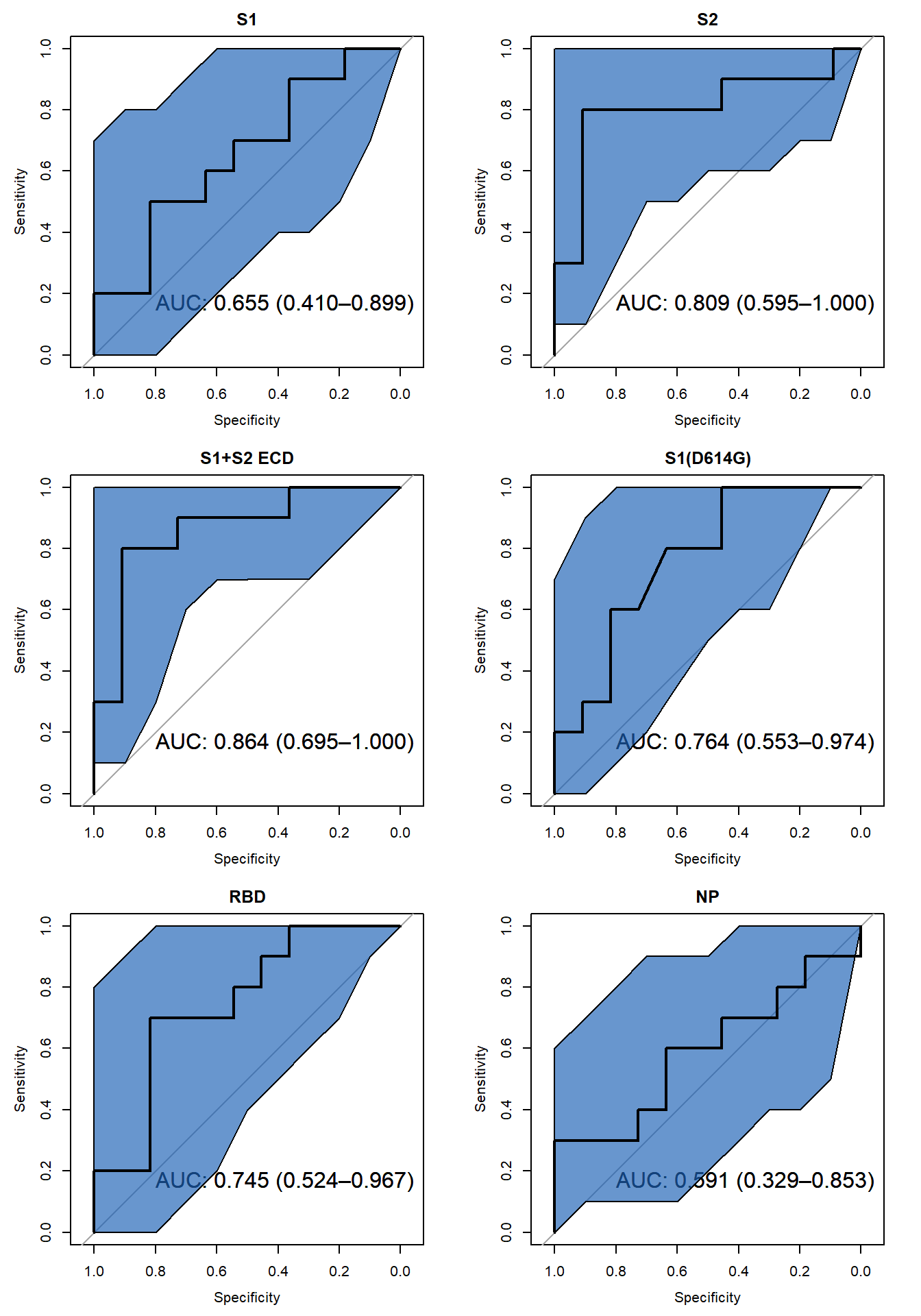


**Fig T** ROC curves for joint model (model 2) to predict death using IgG antibody levels; area under the curve (AUC) is presented within figure with 95% confidence intervals.

## **IgG Covariate Adjusted Analysis**

To examine if the correlation between early antibody levels remains could be explained by confounding due to other risk factors, we fit a covariate adjusted logistic regression model to predict mortality. To build this model, we used a backwards selection approach to identify the most important predictors of mortality. For this purpose, we used data from all 20 subjects who had a Week 1 antibody level measurement. Other than Week 1 antibody level we included in the model Age and binary indicators for Intubation or Ventilation Use, Convalescent Sera or Plasma Use, Diabetes, Hypertension, Kidney Disease, Obesity and Hospitalization within four days of onset. We did not include Week 2 and Week 3 antibody levels as our exploratory analysis indicated no association between these and the outcome. Further, of the 20 subjects, all expired subjects were male, thus the model could not be adjusted for the effect of sex. Below we present the results for models that included Week 1 S1, S2 and RBD IgG antibody level. We fit a separate model for each protein as these measurements were highly correlated. Thus, we aimed to investigate if there are predictors of mortality other than antibody level that could explain the observed association between Week 1 antibody level and mortality, rather than which protein was the most predictive.

Table G. Selected (Using Backwards Selection) Logistic Regression Model to Predict Mortality using S1 Week 1 Antibody Level

|  | | | |
| --- | --- | --- | --- |
|  | Estimate | Std. Error | P-value |
| Intercept | 5.48 | 3.11 | 0.08 |
| Week1 S1 Ab Level | -0.14 | 0.06 | 0.03 |
| Age | -0.07 | 0.05 | 0.13 |
| Kidney Disease | 2.19 | 1.63 | 0.18 |
| Obese or Morbidly Obese | -2.78 | 1.68 | 0.1 |

Table H. Selected (Using Backwards Selection) Logistic Regression Model to Predict Mortality using S2 Week 1 Antibody Level

|  | | | |
| --- | --- | --- | --- |
|  | Estimate | Std. Error | P-value |
| Intercept | 12.73 | 5.69 | 0.03 |
| Week1 S2 Ab Level | -0.36 | 0.16 | 0.02 |
| Age | -0.12 | 0.06 | 0.05 |
| Diabetes | -2.24 | 1.92 | 0.24 |
| Kidney Disease | 3.95 | 2.35 | 0.09 |
| Obese or Morbidly Obese | -4.46 | 2.34 | 0.06 |

Table I. Selected (Using Backwards Selection) Logistic Regression Model to Predict Mortality using RBD Week 1 Antibody Level

|  | | | |
| --- | --- | --- | --- |
|  | Estimate | Std. Error | P-value |
| Intercept | 5.66 | 3.1 | 0.07 |
| Week1 RBD Ab Level | -0.14 | 0.07 | 0.03 |
| Age | -0.07 | 0.05 | 0.11 |
| Kidney Disease | 2.21 | 1.58 | 0.16 |
| Obese or Morbidly Obese | -2.92 | 1.65 | 0.08 |

Logistic Regression models including NP, S1S2ECD and S1D614G proteins failed to converge due to the small sample size. In the above tables we see that the week 1 antibody level remains the best predictor of mortality even after adjusting for other possible covariates in our small sample. With a larger sample we could possibly explore the relationship between antibody level and mortality using daily measurements. It should be noted that here we use the median antibody level in Week 1 for subjects contributing more than measurement. However, we weighted the data to give more weight to subjects contributing more observations. The weights for each subject was proportional to the amount of data points contributed by the subject and such that the weighted sample size was equal to original sample size.

### **Anti-SARS CoV2 IgG Kinetics – Individual Line Plots**

In the following figures we chart the observed IgG antibody titer values (log transformed) for each patient over time after onset. Each line represents a patient in the expired (right panel) or discharged group (left panel). For visual aid, we also include the same figure with a smoothed regression line fit to the observed data and a 95% confidence interval band around this line.


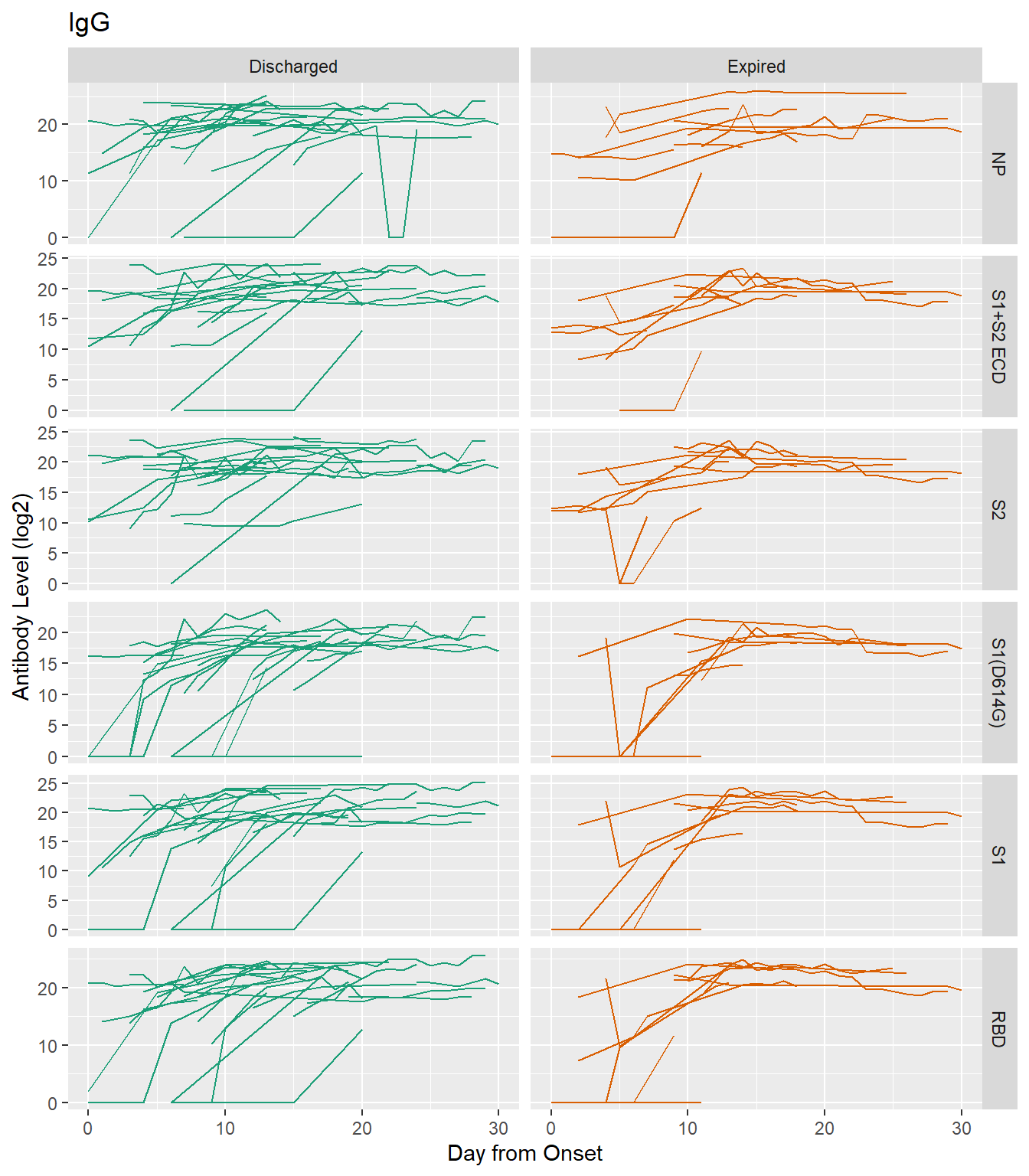


**Fig U Anti-SARS CoV2 IgG Kinetics (Individual).** Observed IgG antibody titer values (log transformed) in expired and discharged groups of patients by protein antigen. Lines represent individual patients


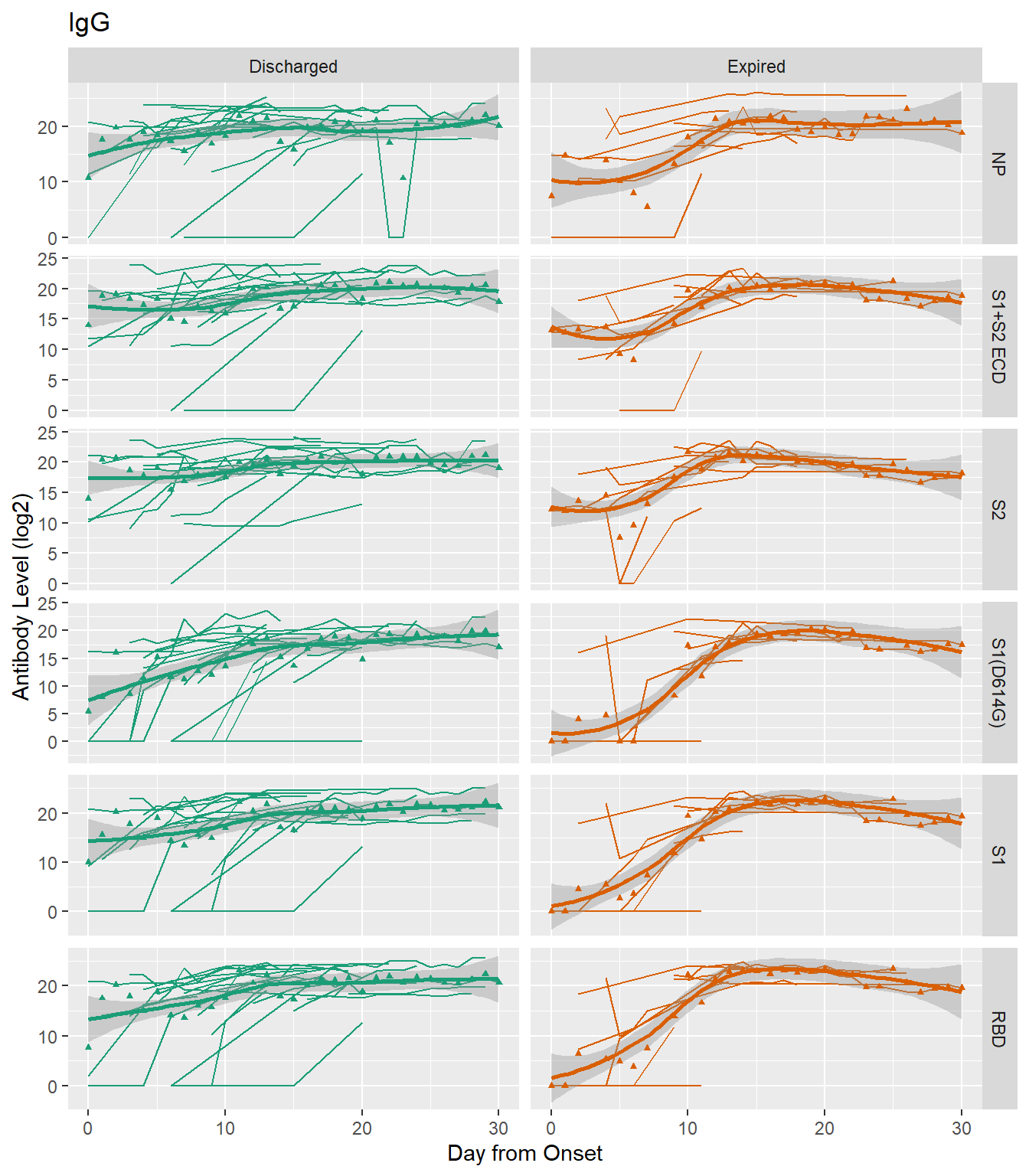


**Fig V Anti-SARS CoV2 IgG Kinetics (Individual) With Smoothed Regression Lines.** Observed IgG antibody titer values (log transformed) in expired and discharged groups of patients by protein antigen. Lines represent individual patients and bolded lines are smoothed regression lines fit to the observed data with 95% confidence intervals

1. Albert PS. A linear mixed model for predicting a binary event from longitudinal data under random effects misspecification. Statist Med. 2012;31(2):143-154. [↑](#footnote-ref-2)
